# Editorial: Novel anti-cancer drugs combination radio-immunotherapy strategy: new frontiers in cancer immunotherapy

**DOI:** 10.3389/fimmu.2025.1747813

**Published:** 2025-11-28

**Authors:** Wesley Burrow, Jonghoon Chang, Ammad Ahmad Farooqi, Shigao Huang, Wensi Tao

**Affiliations:** 1 Department of Medical Education, Dr. Kiran C. Patel College of Allopathic Medicine, Nova Southeastern University, Fort Lauderdale, FL, United States; 2 Institute of Biomedical and Genetic Engineering (IBGE), , Islamabad, Pakistan; 3 Department of Radiation Oncology, Xijing Hospital, The Fourth Military Medical University, Xi’an, China

**Keywords:** immunotherapy, radiotherapy, combined therapy, translational research, tumor microenvironment

Cancer is a heterogeneous and therapeutically challenging disease (1). Mechanistic insights gained through technical developments in single-cell transcriptomics, proteomics, advanced computational models and high-dimensional spatial platforms have enabled basic and clinical researchers to unravel myriad of underlying mechanisms (2, 3). Essentially, accumulating evidence has uncovered mechanisms by which cancers aggressively generate immunosuppressive networks that overcome anti-tumor immunity and confer resistance to cancer immunotherapeutics (4). Immunotherapy, especially when combined with radiotherapy, can enhance cancer treatment by activating the body’s immune system against tumors. However, not all patients benefit (5). Integration of a mechanistic characterization of tumor microenvironment with genetic data will not only empower the design of tailored therapeutic approaches but also improve patient recruitment in early phases of clinical trials.

The field is now moving towards multi-modal approaches that integrate combination therapies to improve outcomes for more people (6). Addressing these unresolved and outstanding questions through translational research and rationally designed trials will be valuable for the improvement of patients with durable clinical outcome. In this Research Topic, we have selected clinically and mechanistically valuable articles to set spotlight on the multi-pronged approaches for cancer treatment.

Zhang et al. investigated P3-GemOx, a novel PD-1–based immunochemotherapy combining pegaspargase, gemcitabine, oxaliplatin, and liposomal mitoxantrone, in patients with relapsed or refractory NK/T-cell lymphoma. Compared with their previously reported PP-GemOx regimen, P3-GemOx demonstrated much higher response rates, as all patients showed responses and most reached CR. The 1-year PFS and OS were 100%, and toxicities were manageable. Their results indicate that the addition of liposomal mitoxantrone significantly improves antitumor efficacy without added toxicity and call for further clinical confirmation of the use of P3-GemOx in aggressive NK/T-cell lymphoma.

Huang et al. assessed the cost-effectiveness of lurbinectedin plus atezolizumab (LU-AT) as a first-line treatment for extensive-stage small-cell lung cancer using a partitioned survival model based on the IMforte trial. Compared with atezolizumab alone, LU-AT yielded a 0.21 QALY gain but was associated with significantly higher costs, resulting in an ICER of $374,167 per QALY in China and $1,071,238 per QALY in the United States, well beyond accepted thresholds. One-way sensitivity analyses demonstrated that the results are stable, with drug price and PFS utility being major cost drivers. Their results concluded that despite clinical benefit, LU-AT is not economically viable at current prices and highlighted the need for price adjustment to enhance affordability.

Shang et al. present an unusual case of a 59-year-old patient with RET fusion–positive non–small cell lung cancer (NSCLC) with brain metastases. The report highlights an exceptional therapeutic response to a combined chemoimmunotherapy regimen (a total of six cycles of pemetrexed plus cisplatin with camrelizumab) and whole-brain radiotherapy (37.5 Gy in 15 fractions). This treatment approach led to complete remission with minimal chemotherapy-related toxicity, highlighting the potential synergistic effect between immunotherapy and radiotherapy in managing advanced NSCLC with intracranial involvement.

Gu et al. are conducting a prospective, single-center, single-arm phase II trial to evaluate the pathological complete response (primary endpoint) to neoadjuvant long-course concurrent chemoradiotherapy, followed by six cycles of CapeOX combined with sintilimab. The study targets patients with mid-to-low locally advanced rectal cancer (LARC) classified as high- or very high-risk, and exhibiting a proficient mismatch repair (pMMR) phenotype.

Huang et al. provided a comprehensive mini-review on the immunoprotective mechanisms and translational potentials of radioprotective agents in cancer radiotherapy. The authors summarize the key compound classes that could lessen radiation-induced oxidative stress, apoptosis, and immune damage: sulfur derivatives, cytokines, antioxidants, and superoxide dismutase mimetics. Emphasis on amifostine and palifermin, as well as nanoparticle and gene therapy-based strategies, shows how immune-integrated radioprotection may improve therapeutic precision and patient outcomes.

Chen et al. performed a retrospective analysis of 23 patients with PD-L1–negative, EGFR/ALK wild-type metastatic NSCLC treated with the novel BRICS regimen (Bifidobacterium probiotics, stereotactic body radiotherapy 24 Gy/3 fractions, low-dose chemotherapy mainly nab-paclitaxel, and PD-1 inhibitors). The study reported ORR 95.7%, DCR 95.7%, median PFS 16 mo and OS 32.7 mo with no ≥ grade 3 toxicities. Their results support a multimodal, immune-oriented sequencing strategy as a potential approach for this patient population.

Cai et al. reviewed the progress made in ultrasound-assisted immunotherapy against malignant tumors, underlining the enhancement of immune activation and drug delivery by ultrasound through its mechanical and cavitation effects. The authors detail how focused ultrasound can increase antigen exposure, promote immune-cell infiltration, and synergize with checkpoint inhibitors and nanocarriers. They also discuss very promising preclinical data and early clinical results showing increased antitumor activity with minimal additional toxicity. This review emphasizes that ultrasound may serve as a noninvasive adjunct with the potential to turn local ablation into systemic immune activation.

Nazarova et al. retrospectively evaluated the combination of SRT with immune checkpoint inhibitors in patients with metastatic uveal melanoma, a malignancy that has been traditionally resistant to systemic therapy. Among 24 patients treated with PD-1 or dual PD-1/CTLA-4 blockade plus SRT, the overall response rate was 39.1%, while the median progression-free survival and overall survival amounted to around 11.6 and 27.6 months, respectively. The regimen was well tolerated, indicating that precision radiotherapy can enhance systemic immunotherapy responses and improve outcomes in metastatic uveal melanoma.

Collectively, these findings reflect an accelerating convergence of precision radiotherapy, immune modulation, and targeted therapy toward more effective and integrated oncologic care.
